# The RNA‐binding protein LARP4 regulates cancer cell migration and invasion

**DOI:** 10.1002/cm.21336

**Published:** 2016-09-26

**Authors:** Shailaja Seetharaman, Ella Flemyng, Jiazhen Shen, Maria R. Conte, Anne J. Ridley

**Affiliations:** ^1^ Randall Division of Cell and Molecular BiophysicsKing's College LondonNew Hunt's House, Guy's CampusLondonSE1 1ULUnited Kingdom

**Keywords:** La‐related proteins, actin cytoskeleton, cell morphology, cancer cells, immunofluorescence

## Abstract

LARP4 is a La‐related RNA‐binding protein implicated in regulating mRNA translation, which interacts with poly(A)‐binding protein (PABP). We previously identified LARP4 in an RNAi screen as one of several genes that regulate the shape of PC3 prostate cancer cells. Here we show that LARP4 depletion induces cell elongation in PC3 cells and MDA‐MB‐231 breast cancer cells. LARP4 depletion increases cell migration and invasion, as well as inducing invasive cell protrusions in 3D Matrigel. Conversely, LARP4 over‐expression reduces cell elongation and increases cell circularity. LARP4 mutations are found in a variety of cancers. Introduction of some of these cancer‐associated mutations, including a truncation mutant, into LARP4 enhances its effects on cell morphology. The truncation mutant shows enhanced interaction with PABP. We propose that LARP4 inhibits migration and invasion of cancer cells, and that some cancer‐associated mutations stimulate these effects of LARP4. © 2016 The Authors. Cytoskeleton Published by Wiley Periodicals, Inc.

**Abbreviations used**
ANOVA: analysis of variance
COSMIC: catalogue of somatic mutations in cancer
PABP: poly(A)‐binding protein
PBM: PABP‐binding motif
TOP: terminal oligopyrimidine tract

## Introduction

Cell migration is essential for animal development and tissue repair, and contributes to human diseases including cancer progression [Friedl and Gilmour, 2009; Ridley, 2015]. Cell migration is initiated by extracellular cues from neighbouring cells or the extracellular matrix. Cell migration and invasion through the extracellular matrix involves dynamic changes to the cytoskeleton, cell–cell adhesions and cell–matrix interaction.

Genome‐wide RNA interference (RNAi) screens have been used to identify putative actin regulators that are not well‐studied for their role in cytoskeletal dynamics. For instance, 99 genes that affected migration of the distal tip cells during gonadogenesis were identified in an RNAi screen in *Caenorhabditis elegans* [Cram et al., 2006]. Similarly, La‐related protein 4 (LARP4) was identified as one of several novel regulators of prostate cancer cell morphology [Bai et al., 2011] based on a previous genome‐wide RNAi screen in *Drosophila melanogaster* [Rohn et al., 2011]. Depletion of LARP4 in PC3 prostate cancer cells resulted in cell elongation, a phenotype similar to that of depleting several other proteins including the Rho GTPases RhoA, RhoU and the formin Dia1. In addition, there was an increase in long thin protrusions containing microtubules in LARP4‐depleted cells [Bai et al., 2011].

LARPs are ancient RNA‐binding proteins (RBPs) which are expressed in all eukaryotes and are subdivided in 5 families: LARP1, La (also known as LARP3), LARP4 (which includes LARP4 and LARP4B in vertebrates), LARP6 and LARP7 [Bousquet‐Antonelli and Deragon, 2009]. LARPs share a common RNA recognition unit termed the La module, consisting of a La motif (LaM) and an adjacent RNA‐recognition motif (RRM1), first discovered in La [Alfano et al., 2004; Bousquet‐Antonelli and Deragon, 2009]. Intriguingly, despite the high sequence conservation in this RNA recognition unit, LARPs differ significantly in their RNA substrate discrimination. For example, whereas La recognises specifically single‐stranded (ss) 3′‐UUU_OH_ stretches, affecting maturation processes of the target RNAs [Kotik‐Kogan et al., 2008; Bayfield et al., 2010], LARP4 has been found to bind to ss oligoA sequences [Bayfield et al., 2010; Yang et al., 2011].

*LARP4* genes are present in some protists and in all animals tested but are absent from plants and yeasts [Merret et al., 2013]. Mammalian LARP4 (also known as LARP4A) has affinity for poly(A) RNA, suggesting it could bind to the poly(A) tail of mRNAs, whereas LARP4B binds to AU‐rich regions in the 3' untranslated regions of mRNAs [Kuspert et al., 2015]. This implies that LARP4 and LARP4B may have distinct functions. LARP4 and LARP4B have also been found to interact with the poly(A)‐binding protein (PABP) and with Receptor for Activated C Kinase (RACK1), a 40S ribosome‐ and mRNA‐associated kinase [Coyle et al., 2009; Schaffler et al., 2010; Yang et al., 2011], consistent with a translation‐related function for LARP4 and LARP4B. Indeed overexpression of human LARP4 resulted in increased mRNA stability whereas knockdown of LARP4 caused a 15‐20% reduction in translation, indicating that LARP4 promotes mRNA stability [Yang et al., 2011]. LARP4 could therefore regulate cell morphology through its binding and translational regulation of mRNAs encoding cytoskeletal regulators. Furthermore, the interaction of LARP4 with RACK1 may be particularly relevant in this context, as RACK1 has been reported to play a role in cell adhesion and migration [Gandin et al., 2013].

Here, we describe the first known cellular phenotype for LARP4. We demonstrate that LARP4 depletion induces cell elongation and increases cell migration speed in both PC3 prostate cancer cells and MDA‐MB‐231 breast cancer cells. Depletion of LARP4 also increased invasion through extracellular matrix. The catalogue of somatic mutations in cancer (COSMIC) reports more than 130 LARP4 mutations in various cancer types. Five cancer‐associated missense mutations and one nonsense mutation (a protein‐truncating stop codon) were introduced into LARP4, several of which enhanced the phenotype induced by LARP4 overexpression. These results indicate that LARP4 regulates cancer cell morphology, migration and invasion, which are key processes in the development of cancers and other diseases.

## Results

### LARP4 Depletion Induces Cell Elongation

**Figure 1:**
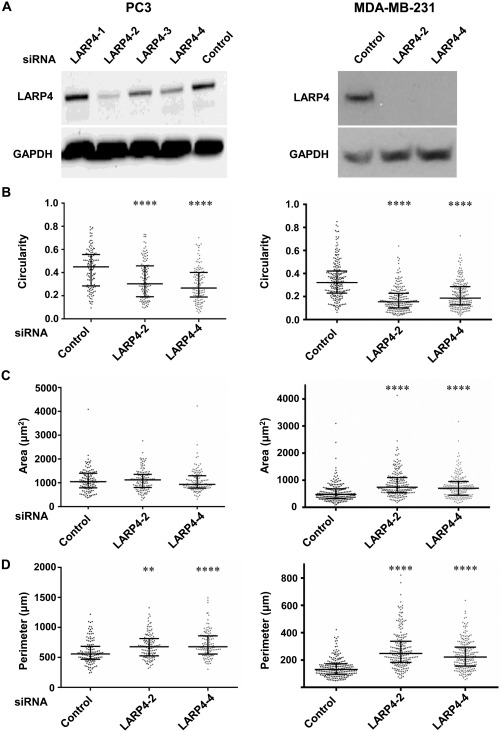
**LARP4 depletion induces cell elongation**. PC3 and MDA‐MB‐231 cells were transfected with control siRNA or the indicated siRNAs targeting LARP4. (**A**) Western blots showing LARP4 protein levels in PC3 cells (left panel) and MDA‐MB‐231 cells (right panel), 72 h after siRNA transfection. GAPDH was used as the loading control. (**B**–**D**) Cell shape parameters, including cell circularity (B), area (µm^2^; C) and perimeter (µm; D) of PC3 cells (left panels) and MDA‐MB‐231 cells (right panels); *n* = 123 (≥33 cells/experiment) from three independent experiments for PC3 cells, and *n* = 213 cells (≥61 cells/experiment) from three independent experiments for MDA‐MB‐231 cells. Values represent the cell shape parameter of each cell as a dot and the median with 25th and 75th percentiles; one‐way ANOVA followed by Tukey's multiple comparisons test; **P < 0.01, ****P < 0.0001.

To study the effects of LARP4 on cell morphology, LARP4 was depleted by siRNA‐mediated knockdown in MDA‐MB‐231 breast cancer cells and PC3 prostate cancer cells, both of which migrate predominantly as single cells and do not express the epithelial cell‐cell adhesion molecule E‐cadherin [Neve et al., 2006; Valderrama et al., 2012]. Our previous studies describing an effect of LARP4 depletion on PC3 cell morphology were carried out using a pool of 4 siRNAs in PC3 cells [Bai et al., 2011]. Two of these siRNAs, LARP4‐2 and LARP4‐4, with good knockdown efficiencies in both PC3 cells and MDA‐MB‐231 cells, were chosen for the remaining experiments (Fig. 1A).

Depletion of LARP4 resulted in cell elongation and long thin protrusions in both PC3 and MDA‐MB‐231 cells (Fig. S1 in Supporting Information). Cell circularity was determined as a measure of cell shape: a circle has a cell circularity value of 1, whereas a line has a circularity value of 0. Cell circularity values were reduced in LARP4‐depleted cells compared to control siRNA transfected cells (Fig. 1B), indicating that LARP4 knockdown results in cell elongation. LARP4 depletion in MDA‐MB‐231 cells increased cell area as compared to control siRNA‐transfected cells (Fig. 1C), although this effect was not observed in PC3 cells. Quantification of cell shape parameters also revealed that the depletion of LARP4 in PC3 and MDA‐MB‐231 cells resulted in a significant increase in cell perimeter as compared to the control cells (Fig. 1D), consistent with the more elongated phenotype. This result is expected based on the increase in cell elongation and area on LARP4 depletion.

### LARP4 Depletion Increases Cell Migration

**Figure 2:**
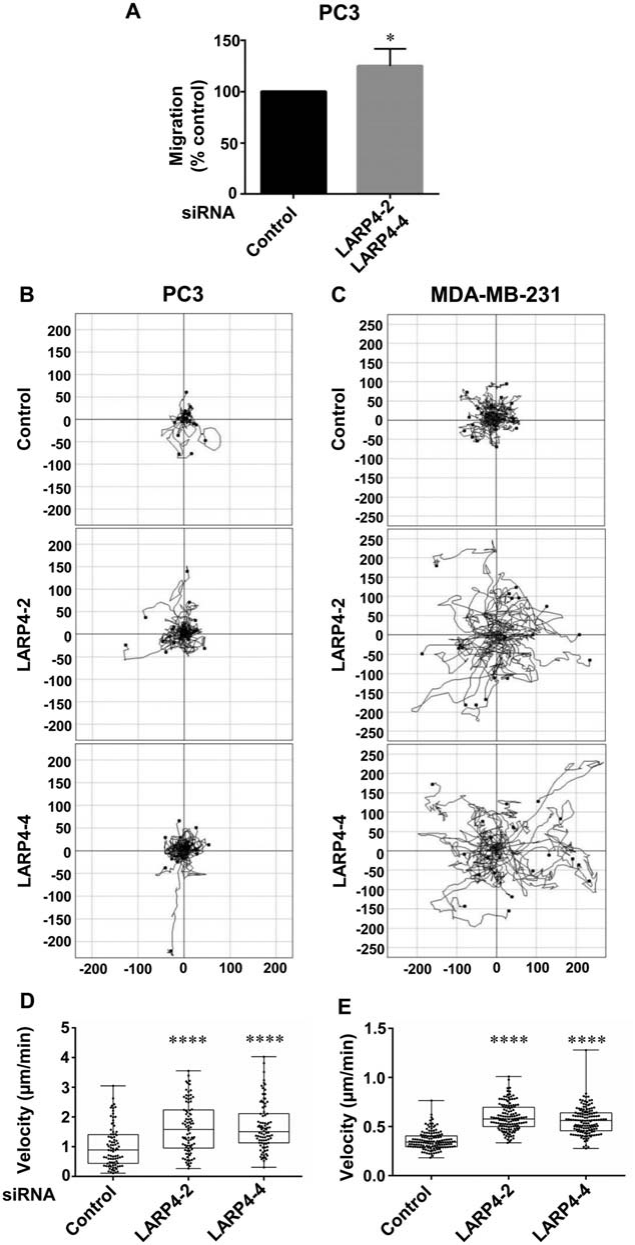
**LARP4 depletion increases cell migration**. (**A**) PC3 cells were transfected with a pool of LARP4 siRNA2 and LARP4 siRNA4 or control siRNA. Cells were seeded onto Matrigel‐coated wells with stoppers. After 24 h, stoppers were removed, and images of the wells were acquired (t = 0) and 24 h later (t = 24). Values represent the mean migration into the gap left by the stopper as a percentage of the control ± SEM; *n* = 3 independent experiments (4 technical replicates/experiment); one‐tailed, two‐sample t‐test; *P < 0.05. (**B**–**E**) PC3 and MDA‐MB‐231 cells were transfected with control siRNA or siRNAs targeting LARP4. Images of cells, 72 h after siRNA transfection, were captured at 1 frame/10 min for 16 h by time‐lapse microscopy. (B–C) Plots show example tracks of 20 PC3 (**B**) or MDA‐MB‐231 cells (**C**) from one of three independent experiments. (D–E) Mean migration speed (μm/min) of PC3 (**D**) and MDA‐MB‐231 cells (**E**); *n* = 88 cells (≥ 18 cells per experiment) from three independent experiments for PC3 cells, and *n* = 142 (≥44 cells per experiment) from three independent experiments for MDA‐MB‐231 cells. Values represent the migration speed of each cell as a dot and the median with 25th and 75th percentiles; one‐way ANOVA followed by Tukey's multiple comparisons test; ****P < 0.0001.

To investigate whether the changes in cell morphology caused by LARP4 depletion might affect cell migration, LARP4 was depleted in PC3 and MDA‐MB‐231 cells by siRNA‐mediated knockdown, and cell migration followed. First, a modified wound healing migration assay was carried out, in which cells migrate into a cell‐free gap created by removing a stopper (see Materials and Methods). The migration of LARP4‐depleted PC3 cells into the cell‐free gap was significantly higher as compared to the control cells (Fig. 2A). Second, cell migration was followed by time‐lapse microscopy (Movies S1 and S2 in Supporting Information). By tracking the migratory paths of PC3 and MDA‐MB‐231 cells (Figs. 2B and 2C) in a random motility assay, LARP4‐depleted cells migrated further than control siRNA‐transfected cells. LARP4 depletion resulted in a significant increase in cell migration speed (Figs. 2D and 2E). Changes in cell morphology on LARP4 depletion therefore correlate with an increase in migration speed.

### LARP4 Mutations Do Not Affect LARP4 Localization or Impair Binding to the Poly(A)‐Binding Protein

**Figure 3:**
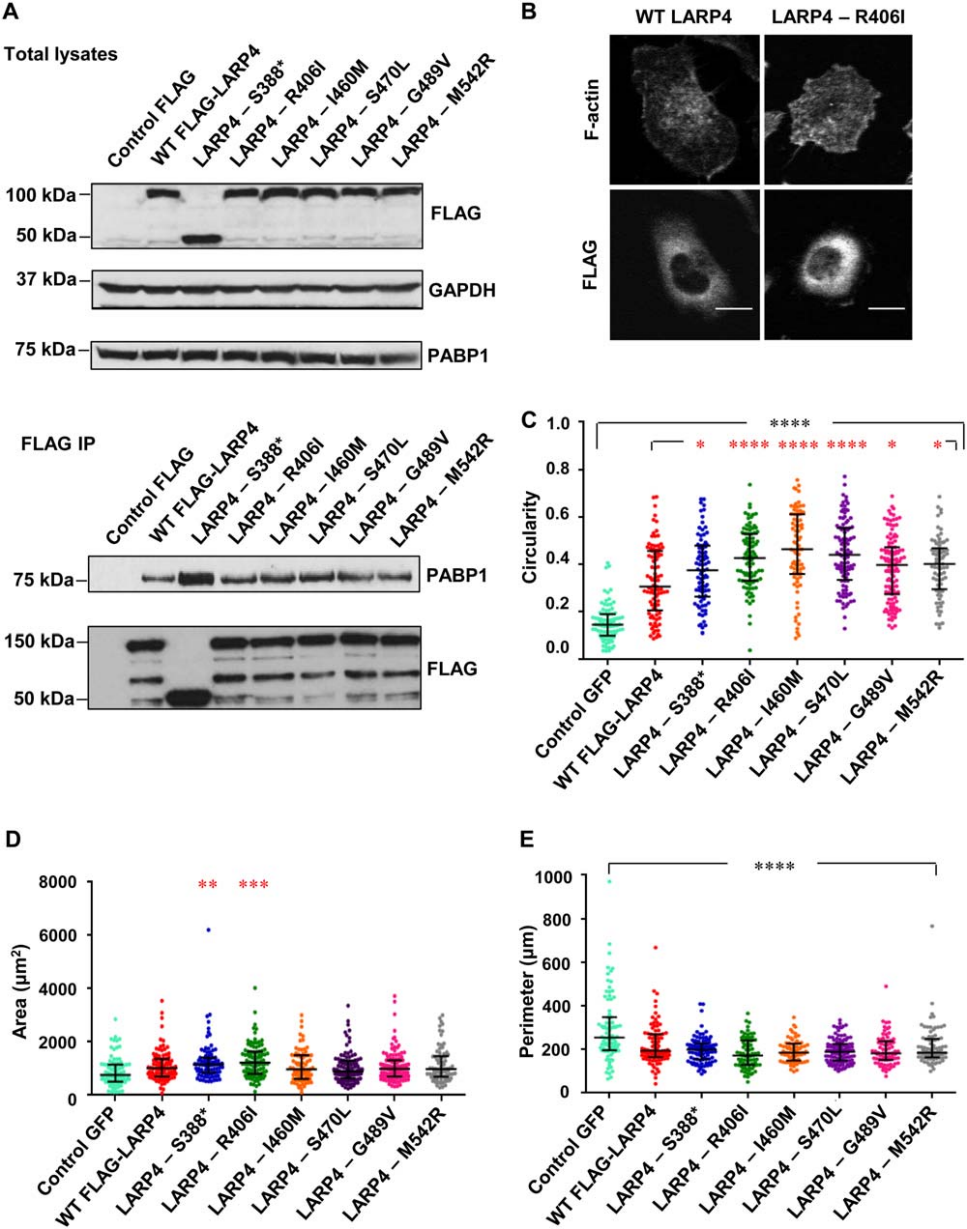
**LARP4 and LARP4 cancer‐associated mutants interact with PABP and increase cell circularity**. (**A**) HEK293T cells were transfected with empty pCMV2‐FLAG or vectors encoding WT LARP4 or LARP4 mutants. After 24 h, cells were lysed and immunoprecipitated with anti‐FLAG antibody‐conjugated agarose beads. Immunoprecipitates and total cell lysates were immunoblotted with the indicated antibodies. GAPDH was used as a loading control. Blots are representative of two independent experiments. (**B**) Representative images for the cytoplasmic localization of LARP4 and LARP4‐R406I. Cells were stained for F‐actin and FLAG epitope. Scale bars: 15 µm. (**C**–**E**) Cell circularity, area and perimeter of WT LARP4‐expressing or LARP4 mutant‐expressing cells as compared to control GFP; *n* = 84 cells from three independent experiments (≥24 cells per experiment). Values represent the cell shape parameter of each cell as a dot and the median with 25th and 75th percentile; one‐way ANOVA followed by Tukey's multiple comparisons test; **P <0.01, ***P <0.001, ****P <0.0001 compared to WT‐LARP4 (* in red) or to control (* in black). [Color figure can be viewed at wileyonlinelibrary.com]

The *LARP4* gene is located on human chromosome 12q13.12. Over 130 *LARP4* mutations in cancers are reported in the COSMIC. Of these, six mutations (present in COSMIC on 7 October 2014) were chosen that are located in the C‐terminal region of LARP4, a part of the protein that appears to mediate interactions with protein partners of LARP4 and LARP4B such as RACK1 and PABP [Schaffler et al., 2010; Yang et al., 2011]. The following amino acid substitutions, S388*, R406I, I460M, S470L, G489V and M542R, were created in the *LARP4* cDNA by site‐directed mutagenesis (Fig. S2A in Supporting Information). To determine whether any of the mutations affected protein localization or stability, LARP4 was overexpressed in PC3 cells by transfecting DNA encoding wild‐type FLAG‐epitope tagged LARP4 or each of the mutant LARP4 proteins. The WT LARP4 and mutant constructs expressed in PC3 cells were approximately 80 kDa in size (Fig. 3A; LARP4 has 725 amino acids). LARP4‐S388* has a stop codon instead of S388, lacks amino acids 388 to 725 and therefore had a lower molecular weight (Fig. 3A). None of the LARP4 mutations affected protein stability. In agreement with previous studies using HeLa cells [Yang et al., 2011], we found that FLAG‐LARP4 localizes predominantly in the cytoplasm of PC3 cells (Fig. 3B). The LARP4 mutations tested did not alter LARP4 localization (Fig. 3B, Fig. S2B in Supporting Information).

Two regions of LARP4 have been reported to contribute to binding to PABP: an N‐terminal PAM2 motif and a putative PABP‐binding motif (PBM) mapping between amino acids 287 and 429 [Yang et al., 2011]. Two of the mutants (S388* and R406I) map to the PBM, and we therefore tested whether any of the LARP4 mutants had altered interaction with PABP. All of the mutants co‐immunoprecipitated with PABP1, and interestingly the LARP4‐S388* truncation mutant showed stronger interaction with PABP1 compared to the other mutants or wild‐type LARP4 (Fig. 3A). This is consistent with previous results showing that some LARP4 deletion mutants lacking the C‐terminal region (e.g 1‐430 and 1‐504) show increased interaction with PABP compared to wild‐type LARP4 [Yang et al., 2011], which suggests the C‐terminal region contains inhibitory sequences for PABP binding.

### LARP4 Mutations Differentially Affect Cell Shape

The effect of overexpressing wild‐type LARP4 and LARP4 mutants on cell morphology was investigated in PC3 cells. Expression of wild‐type LARP4 increased cell circularity (Fig. 3C), consistent with our observation that LARP4 depletion reduced circularity and increased elongation. Expression of LARP4 mutants in PC3 cells resulted in an increase in cell circularity as compared to the control GFP‐expressing cells, and all of the LARP4 mutants further increased cell circularity compared to wild‐type LARP4 (Fig. 3C). Two of the LARP4 mutants, R406I and S388*, significantly increased cell area when compared to wild‐type LARP4, although wild‐type LARP4 did not change cell area significantly compared to control GFP‐expressing cells (Fig. 3D). The expression of all LARP4 proteins resulted in a significant decrease in cell perimeter as compared to control cells (Fig. 3E), opposite to the effect of LARP4 depletion (Fig. 1). The slight increase in cell area and decrease in cell perimeter are consistent with LARP4 overexpression inducing a more regular circular shape compared to control cells. Indeed, cell circularity is proportional to cell area and inversely proportional to the square of the perimeter (see Materials and Methods). Overall, these data indicate that several cancer‐associated mutations in LARP4 enhance its effects on cell shape, predominantly by reducing cell elongation and increasing circularity, which would be predicted to reduce migration and invasion based on our previous studies with PC3 cells [Vega et al., 2011]. This suggests that some of the mutants could be more active than wild‐type LARP4.

### LARP4 Depletion Promotes Invasion

**Figure 4:**
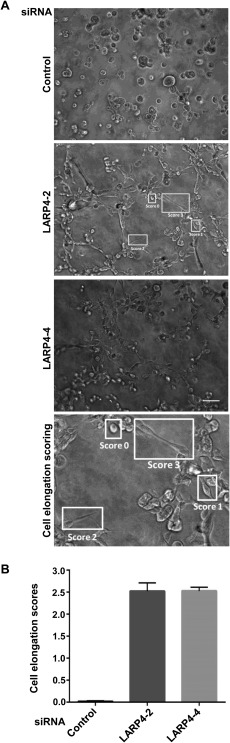
**LARP4 depletion causes cell elongation in 3D**. MDA‐MB‐231 cells were transfected with control siRNA or siRNAs targeting LARP4. (**A**) 3D morphology assay in Matrigel. 48 h after siRNA transfection, cells were embedded in Matrigel and seeded onto Matrigel‐coated 96‐well plates. Example images are shown. Scale bar: 100 μm. (**B**) Cells were assigned a shape score from 0 to 3, as indicated on the image for LARP4‐2 siRNA and in the enlarged image below (Cell elongation scoring): a score of 0 indicates a round cell and a score of 1, 2 or 3 indicates cells with progressively longer protrusions. Graph shows mean elongation scores of cells for each condition +/‐ SEM; *n* =3 independent experiments (4 images per experiment per condition).

Cancer cells often have different morphologies in 3D as compared to 2D [Doyle et al., 2013]. In order to investigate whether LARP4 contributes to cell elongation in 3D as well as on 2D substrata, a 3D morphology based assay was carried out using MDA‐MB‐231 cells embedded in Matrigel [Colomba and Ridley, 2014], which is a cell‐derived extracellular matrix used to mimic the environment of tumours [Gill and West, 2014]. The LARP4‐depleted cells had a striking elongated phenotype in Matrigel as compared to the control siRNA transfected cells (Fig. 4A). Most LARP4‐depleted cells were highly elongated, whereas nearly all control cells were rounded (Fig. 4B). The morphology of LARP4‐depleted cells in 3D is therefore similar to that in 2D.

**Figure 5:**
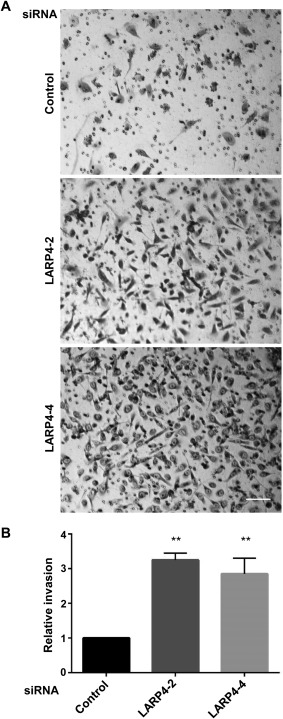
**LARP4 depletion increases invasion**. PC3 cells were transfected with control siRNA or siRNAs targeting LARP4. After 48 h, cells in 0.1% FCS containing RPMI were seeded on the top chamber of transwells and RPMI containing 1% FCS was added to the bottom wells. (**A**) Representative images of cells that migrated to the bottom of the transwell. Cells were fixed with 70% ethanol and stained with 0.2% crystal violet solution. Scale bar: 100 μm. (**B**) Relative invasion index compared to siControl; cells on the bottom of each transwell were counted in 10 images per experiment per condition (5 images per technical replicate), *n* = 3 independent experiments. Values represent fold‐increase ± SEM of invaded cells compared to control siRNA; one‐way ANOVA followed by Tukey's multiple comparisons test, **P <0.01.

To test whether LARP4 affected cancer cell invasion, PC3 cell invasion through Matrigel‐coated transwells was analysed (Fig. 5A) [Vega et al., 2011]. Approximately 3‐fold more LARP4‐depleted cells invaded through the transwells than control siRNA‐transfected cells (Fig. 5B). This increased invasive behaviour of LARP4‐depleted cells correlates with the elongated and protrusive phenotype observed in 2D as well as in 3D.

## Discussion

In this study, we show that LARP4 affects cancer cell morphology, migration and invasion. The overexpression of LARP4 increased cell circularity, whereas LARP4 depletion resulted in cell elongation. We also found that LARP4 suppresses migration, as the migration speed of two cancer cell lines that migrate predominantly as single cells, PC3 and MDA‐MB‐231, increased upon LARP4 depletion. In addition to the effect of LARP4 on cancer cells in 2D, LARP4 depletion resulted in cell elongation in 3D Matrigel and increased invasion. We therefore conclude that LARP4 alters shape, migration and invasion of PC3 and MDA‐MB‐231 cancer cells.

Cancer cells often have different morphologies in pliable 3D extracellular matrices as compared with hard 2D surfaces [Sahai, 2005; Doyle et al., 2013]. However, it has been previously shown that RhoA depletion in PC3 cells resulted in cell elongation with thin protrusions in 2D as well as 3D environments [Vega et al., 2011]. Similarly, the morphology of LARP4‐depleted MDA‐MB‐231 cells embedded in a thick layer of 3D Matrigel was similar to that in 2D: cells had an elongated phenotype with long thin protrusions under both conditions. The elongated phenotype of LARP4‐depleted cells with protrusions correlates with their increased invasion. Depletion of other proteins, including the Rho GTPases RhoA and RhoU and the formin mDia1, induces a similar phenotype to LARP4‐depleted cells, and, similar to LARP4 depletion, RhoA knockdown increases invasion [Bai et al., 2011; Vega et al., 2011]. On the other hand, the increase in circularity of LARP4‐overexpressing cells resembles that of RhoC or radixin depletion, which inhibit PC3 cell migration and/or invasion [Vega et al., 2011; Valderrama et al., 2012]. We would therefore expect that LARP4‐overexpressing cells would have a reduced ability to migrate or invade. It is probable that LARP4 alters cell phenotype, migration and invasion by altering the expression of proteins that affect these processes, such as components of Rho signaling networks [Ridley, 2015].

Our analysis of LARP4 mutants revealed that all of them increased cell circularity compared to wild‐type LARP4, however R406I, I460M and S470L had the strongest effect. LARP4 R406I and S388* mutants increased cell area compared to wild‐type LARP4. These results suggest that four of the LARP4 cancer‐associated mutations, R406I, I460M, S470L and S388*, may affect cancer cell migration as they elicit greater changes in cell shape compared to wild‐type LARP4. Since LARP4 depletion increases PC3 and MDA‐MB‐231 cell elongation and migration, it can be hypothesized that these LARP4 mutants may inhibit cell migration.

What might be the mechanism to explain the observed phenotype of LARP4? None of the LARP4 mutations analysed in this study (S388*, R406I, I460M, S470L, G489V, M542R) maps onto the RNA‐binding region, which localizes to the protein's N‐terminal half and encompasses the La module [Yang et al., 2011]. In addition to RNA, two proteins, PABP and RACK1, have been identified as direct binding partners for LARP4 and LARP4B, and we therefore investigated whether the LARP4 mutations affected interaction with either of these proteins [Schaffler et al., 2010; Yang et al., 2011]. Our mutational analysis provides new information on LARP4 interaction with PABP: while none of the mutations reduces PABP interaction, the truncation mutant LARP4‐S388* shows enhanced binding to PABP. This indicates that the second putative PBM, mapped previously to residues 287 to 429 [Yang et al., 2011], maps N‐terminal to S388. It will be interesting to test whether PABP contributes to the increased cell spread area induced by this mutant. RACK1 interaction is likely to be within the C‐terminal 430 amino acids of LARP4B, but its site of interaction has not been defined [Schaffler et al., 2010]. All of the LARP4 mutants appear to interact with RACK1 (unpublished data), and thus it is unlikely that loss of RACK1 interaction is responsible for the morphological changes we observe. Further studies are therefore needed to understand the basis for LARP4 responses and the effects of some of its mutants, which may alter interactions with as‐yet‐unidentified partners for LARP4.

*LARP4* mutations are very rare in cancers compared to well‐known mutated genes such as *K‐Ras* or *p53*. The majority of the many somatic mutations present in each cancer probably do not contribute significantly to cancer development, whereas a small subset, called driver mutations, confer clonal selective advantage on the cancer cells and are selected during the evolution of cancer [Nussinov and Tsai, 2015; Pon and Marra, 2015]. The remaining somatic mutations that do not confer growth advantage to cancer cells are known as passenger mutations. It may be that the rare *LARP4* mutations are passenger mutations. However, it has recently been proposed that individual cancers have latent or rare driver mutations that contribute to cancer progression [D'Antonio and Ciccarelli, 2013; Nussinov and Tsai, 2015] We speculate that the *LARP4* mutants which resulted in significant changes in cancer cell morphology could be rare driver mutations, contributing to tumour progression in some human cancers, in concert with known driver mutations.

Of the LARPs, La, LARP1 and LARP7 have also been implicated in cancer, albeit with rather distinct mechanisms. The involvement of La has been thus far associated with its ability to interact with internal ribosome entry sites of genes associated with tumour onset and malignancy (e.g. MDM2, cyclin D1) [Sommer et al., 2011a; Trotta et al., 2003]. Interestingly, La has been reported to stimulate migration and invasion [Sommer et al., 2011b], which is the opposite of what we observe for LARP4. LARP1 is overexpressed in the majority of epithelial malignancies compared to their adjacent normal tissues [Stavraka and Blagden, 2015]. LARP1 may act to promote cancer by its translational regulation of 5' terminal oligopyrimidine tract (TOP)‐containing mRNAs such as mTOR [Mura et al., 2015]. In contrast to La and LARP1, LARP7 is lost or mutated in a number of cancers. LARP7's role as a tumour suppressor is attributed to its function as an inhibitor of the positive transcription factor P‐TEFb, a cyclin‐dependent kinase required for RNA polymerase II transcription elongation [Ji et al., 2014; Stavraka and Blagden, 2015].

The exact mechanism of action of LARP4 in altering cancer cell morphology, migration and invasion is yet to be determined. Our results suggest the intriguing possibility that LARP4 might alter the translation of mRNAs that include migratory regulators. Alternatively LARP4 may act as a global RNA chaperone contributing to the stabilisation and/or modulation of several mRNAs [Hussain et al., 2013], and thus LARP4 depletion would alter the transcriptomic profile leading to cell motility changes. In either case, our results with LARP4 are consistent with a growing body of evidence indicating a prominent role for RBPs in both the development and progression of cancer [Wurth and Gebauer, 2015].

## Materials and Methods

### Cell Lines and Reagents

MDA‐MB‐231 breast cancer cells were grown in DMEM supplemented with 25 mM HEPES and 2 mM glutamine, 1% sodium pyruvate, 10% FCS, 100 µg/ml streptomycin and 100 U/ml penicillin. PC3 prostate cancer cells were cultured in RPMI containing 25 mM HEPES and 2 mM glutamine, 10% FCS, 100 µg/ml streptomycin and 100 U/ml penicillin. The following antibodies were used: FLAG (F7425, Sigma‐Aldrich), LARP4 (gift from Richard Maraia, National Institutes of Health, Maryland), PABP1 (4992, Cell Signaling Technologies), GAPDH (MAB374, Merck Millipore). Mouse anti‐FLAG A2220 (M2, Sigma‐Aldrich) antibody bound to agarose was used for immunoprecipitations. Secondary HRP‐labeled antibodies (anti‐mouse and anti‐rabbit) were from GE Healthcare. Protease and phosphatase cocktail inhibitor tablets were from Roche and Calbiochem.

### DNA Constructs and siRNAs

pCMV2‐FLAG‐LARP4 (human LARP4) was a gift from Dr. Richard Maraia, National Institutes of Health, Maryland. Six LARP4 point mutations reported in cancers (Catalogue of Somatic Mutations in Cancer; 7 October 2014) were incorporated into pCMV2‐FLAG‐LARP4 using QuikChange II XL Site‐directed mutagenesis kit (Agilent Technologies). Primer sequences for mutagenesis were:

S388*: 5′‐TGGTGGTTCAGAACACTAAACAGAGGGCTCTGTAT‐3′.

R406I: 5′‐GTTGAACAGATATAGTTCAATAAACTTTCCAGCTGAACGGC‐3′,

I460M: 5′‐ACGACGAGAAGATGACAGGATGTCAAGACCTCATC‐3′,

S470L: 3'‐TGGAGTAGGAAGTTGTCGACTTAATTTCCGAGGTTGT‐5′,

G489V: 3′‐GTTTAAAAGGTGGAAATGGACATTCAAGTAGTTCTTACGGTCC‐5′,

M542R: 5′‐ACTTCTGCCCAGCAACTCAATAGGAGTACCAGTTC‐3′,

All siRNAs were from Sigma‐Aldrich and Dharmacon (GE Healthcare): siLARP4‐1 (CAUAAGCGUUGUAUUGUAA), siLARP4‐2 (UAGGAUGUCUGAUGUUGUU), siLARP4‐3 (CAAGGGCUAGUAAGGAUUA), siLARP4‐4 (GGACAGUUGAACAGAUAUA), non‐silencing siControl (UUCUCCGAACGUGUCACGU).

### Transfection

For overexpression of LARP4, cells were transfected with pEGFP‐C1 or pCMV2‐FLAG empty vector (controls), or pCMV2‐FLAG‐LARP4 (wild‐type or mutants) using Lipofectamine 2000 (ThermoFisher Scientific) in growth medium without antibiotics. For depletion of LARP4, cells seeded on plates pre‐coated with collagen I (50 μg/ml) were transfected with siControl or siRNAs targeting LARP4 using Oligofectamine (ThermoFisher Scientific) in growth medium without antibiotics. In both cases, transfection medium was replaced with complete medium 6 h after transfection. Of the 4 siRNAs tested, two of them with good knockdown efficiencies, LARP4‐2 and LARP4‐4, were used for further experiments.

### Immunoblotting

Cells were lysed 24 h after transfecting LARP4 cDNA or 72 h after siRNA transfection in lysis buffer (50 mM Tris‐HCl, pH 8.0, 250 mM NaCl, 1% Triton X‐100, 25 mM NaF and 2 mM Na_3_VO_4_) supplemented with protease cocktail inhibitors. Lysates were resolved in 4‐12% polyacrylamide gels (Invitrogen, ThermoFisher Scientific), transferred to nitrocellulose membranes and incubated with antibodies. Membranes were blocked in 5% dried milk powder in Tris‐buffered saline with 0.1% Tween‐20 (TBS‐T) and incubated with the primary antibody for 16‐18 h (except GAPDH for 1 h) at 4°C. Following incubation with primary antibody, membranes were washed 3 times with TBS‐T and incubated with species‐appropriate horseradish peroxidase‐conjugated secondary antibodies.

### Immunoprecipitation

HEK293T cells were lysed 24 h after DNA transfection in 20 mM Tris‐HCl (pH 8.0), 130 mM NaCl, 1% Triton X‐100, and 1 mM Dithiothreitol (DTT), supplemented with protease and phosphatase cocktail inhibitors. After centrifugation, some supernatant was kept for analysis of total lysates, and the remaining supernatants were incubated with anti‐FLAG antibody‐conjugated agarose beads (20 μl for each condition) or 2 h at 4°C. The beads were washed extensively with lysis buffer. Samples were analysed by western blotting as described above.

### Immunofluorescence and Confocal Microscopy

Cells on coverslips were fixed with 4% paraformaldehyde, permeabilized with 0.1% Triton X‐100 and blocked with 3% BSA in PBS. F‐actin, DNA and microtubules were visualized using AlexaFluor 546‐conjugated phalloidin (1:400; Molecular Probes), DAPI (1:10000; Molecular Probes) and/or FITC‐labelled anti‐α‐tubulin (1:1000; Sigma‐Aldrich, clone DM1A). Coverslips were mounted onto glass slides using fluorescence mounting medium (Dako). Images were acquired using a LSM 510 confocal microscope (Zeiss) using a 20X objective and Zen software (Zeiss). Images are from cells fixed 24 h after transfection of DNA encoding LARP4 or its mutants, or 72 h after siRNA transfection.

### Cell Morphology Analysis

Morphological analysis was carried out using ImageJ (National Institutes of Health). The edges of cells were traced using the F‐actin staining boundary. For cells transfected with pCMV2‐FLAG‐LARP4 or its mutants, LARP4‐expressing cells/LARP4‐mutant expressing cells (detected with anti‐FLAG antibody) were quantified and compared to cells transfected with pEGFP‐C1 (control). Boundaries of cells were determined and cell shape parameters including cell area, perimeter and circularity were generated using ImageJ. Circularity is calculated as $4\pi \;\times \;\frac{Area}{{\left(Perimeter\right)}^{2}},$ with a value of 1 indicating a perfect circle and values closer to zero indicating an elongated phenotype of cells. Results were graphically represented using GraphPad Prism 6.

### Cell Migration Assays

For the modified scratch wound migration assay, PC3 cells were transfected with a pool of LARP4 siRNA2 and LARP4 siRNA4 or control siRNA. After 24 h, cells were incubated with Cell Tracker Orange CMRA (1:400; ThermoFisher Scientific) in growth medium for 40 minutes at 37°C, 5% CO_2_. Following incubation, the dye was removed and cells were washed in PBS. Cells were detached, counted and seeded at a density of 4 × x10^4^ cells per well in medium containing 1% FCS in a 96 well plate with Oris^TM^ cell seeding stoppers (Platypus Technologies). Wells were pre‐coated with 100 μg/ml Matrigel for 1 h (BD Biosciences). After 24 h, stoppers were removed, and images of the wells were acquired using a Nikon TE2000‐E microscope with a Plan Fluor 4x objective (Nikon) and a Hamamatsu Orca‐ER digital camera using Volocity software (PerkinElmer). 24 h after acquiring the initial images, wells were imaged again in the same positions to analyse the movement of cells into the cell‐free gap. Image analysis was carried out using ImageJ.

For random cell migration, PC3 cells were seeded at 10^4^ cells per well on Matrigel‐coated 6‐well plates, and MDA‐MB‐231 cells at 2 × 10^4^ cells per well on collagen I‐coated 24‐well plates. Cells were starved in medium containing 0.1% FCS, 48 h after siRNA transfection. After a further 24 h, the medium was replaced with medium containing 1% FCS, immediately before the start of the migration assay. Images were acquired for 16 h at 1 frame/10 min at 37°C using a 10X/0.3 NA Plan Fluor objective on a Nikon TE‐2000 time lapse microscope with a Hamamatsu Orca‐ER camera and Volocity (for PC3 cells) or Micro‐Manager software (for MDA‐MB‐231 cells). Images were acquired from at least two wells each for control siRNA, LARP4‐2 and LARP4‐4. Cells were tracked using ImageJ (Plugin: Manual tracking) to obtain migration speed (μm/min). Cells that died, divided, or moved out of the frame were excluded from the analysis and tracking. The path of each cell was obtained as a track using ImageJ (Plugin: Chemotaxis tool).

Position‐to‐position and well‐well migration parameters were compared to check if the data for each condition were consistent within each experiment and between three different experiments. There were no significant variations between the technical replicates or experimental replicates of each condition indicating that the three independent experiments were consistent.

### 3D Morphology Assay

Flat bottom plates (96‐well) were coated with 7.5 mg/ml Matrigel and incubated at 37°C for 2 h. Cells were embedded in Matrigel 48 h after siRNA transfection: 5x10^4^ MDA‐MB‐231 cells in DMEM without FCS were added to wells of “V” bottom 96‐well plates and mixed with 100 µl of 7 mg/ml Matrigel in DMEM without FCS. This mixture was then added to the Matrigel pre‐coated plates and the Matrigel was allowed to polymerize for 2 h before DMEM without FCS was added on the top. Plates were incubated at 37°C for 24 h.

Four random fields per well were acquired 72 h after siRNA transfection, using a 10X/0.3 NA Plan Fluor objective on a Nikon TE‐2000 time lapse microscope with a Hamamatsu Orca‐ER camera and Micro‐Manager software. Each cell was given a score of 0, 1, 2 or 3, where 0 indicates a round cell, and 1, 2 and 3 indicate cells with progressively longer protrusions. Out‐of‐focus cells were not scored.

### Transwell‐Based Invasion Assay

The transwell‐based BD Biocoat Invasion System (8 µm pore diameter; BD Biosciences) was used for invasion through a Matrigel layer. 48 h after siRNA transfection, 10^4^ PC3 cells in medium containing 0.1% FCS were added to the transwells and medium containing 1% FCS was used as an attractant in the lower chamber. After 24 h, cells on the bottom of the coated transwell were fixed with 70% ethanol and stained with 0.2% crystal violet solution. Random images from three independent experiments (10 per experimental condition) were acquired using a Nikon Eclipse TS100 microscope with a 10× objective and cells counted using ImageJ (Plugin: Cell counter). The cells on the bottom of each transwell were counted. For each experimental condition, the total number of cells from the 10 images was divided by the total number of cells for control siRNA‐transfected cells.

### Statistical Analysis

One‐way analysis of variance (ANOVA) followed by Tukey's multiple comparisons test was used unless stated otherwise. All plots show median with 25^th^ and 75^th^ percentiles unless stated otherwise. Statistical significance is indicated by *P < 0.05, **P < 0.01, ***P < 0.001, ****P < 0.0001 as compared to the control unless stated otherwise.

## Contributions

The project was conceived by A.J.R. and M.R.C. S.S., J.S., and E.F. carried out experiments, and all authors analysed and interpreted the data. S.S. and A.J.R wrote the manuscript, with input from E.F., J.S., and M.R.C.

## Supporting information

Supporting Figures

Supporting Legends

Supporting Movie S1

Supporting Movie S2

## References

[cm21336-bib-0001] Alfano C , Sanfelice D , Babon J , Kelly G , Jacks A , Curry S , Conte MR. 2004 Structural analysis of cooperative RNA binding by the La motif and central RRM domain of human La protein. Nat Struct Mol Biol 11:323–329. 15004549 10.1038/nsmb747

[cm21336-bib-0002] Bai SW , Herrera‐Abreu MT , Rohn JL , Racine V , Tajadura V , Suryavanshi N , Bechtel S , Wiemann S , Baum B , Ridley AJ. 2011 Identification and characterization of a set of conserved and new regulators of cytoskeletal organization, cell morphology and migration. BMC Biol 9:54. 21834987 10.1186/1741-7007-9-54 PMC3201212

[cm21336-bib-0003] Bayfield MA , Yang R , Maraia RJ. 2010 Conserved and divergent features of the structure and function of La and La‐related proteins (LARPs). Biochim Biophys Acta 1799:365–378. 20138158 10.1016/j.bbagrm.2010.01.011 PMC2860065

[cm21336-bib-0004] Bousquet‐Antonelli C , Deragon JM. 2009 A comprehensive analysis of the La‐motif protein superfamily. RNA 15:750–764. 19299548 10.1261/rna.1478709 PMC2673062

[cm21336-bib-0005] Colomba A , Ridley AJ. 2014 Analyzing the roles of Rho GTPases in cancer cell migration with a live cell imaging 3D‐morphology‐based assay. Methods Mol Biol 1120:327–337. 24470035 10.1007/978-1-62703-791-4_21

[cm21336-bib-0006] Coyle SM , Gilbert WV , Doudna JA. 2009 Direct link between RACK1 function and localization at the ribosome in vivo. Mol Cell Biol 29:1626–1634. 19114558 10.1128/MCB.01718-08 PMC2648249

[cm21336-bib-0007] Cram EJ , Shang H , Schwarzbauer JE. 2006 A systematic RNA interference screen reveals a cell migration gene network in C. elegans. J Cell Sci 119:4811–4818. 17090602 10.1242/jcs.03274

[cm21336-bib-0008] D'Antonio M , Ciccarelli FD. 2013 Integrated analysis of recurrent properties of cancer genes to identify novel drivers. Genome Biol 14:R52. 23718799 10.1186/gb-2013-14-5-r52 PMC4054099

[cm21336-bib-0009] Doyle AD , Petrie RJ , Kutys ML , Yamada KM. 2013 Dimensions in cell migration. Curr Opin Cell Biol 25:642–649. 23850350 10.1016/j.ceb.2013.06.004 PMC3758466

[cm21336-bib-0010] Friedl P , Gilmour D. 2009 Collective cell migration in morphogenesis, regeneration and cancer. Nat Rev Mol Cell Biol 10:445–457. 19546857 10.1038/nrm2720

[cm21336-bib-0011] Gandin V , Senft D , Topisirovic I , Ronai ZA. 2013 RACK1 function in cell motility and protein synthesis. Genes Cancer 4:369–377. 24349634 10.1177/1947601913486348 PMC3863339

[cm21336-bib-0012] Gill BJ , West JL. 2014 Modeling the tumor extracellular matrix: Tissue engineering tools repurposed towards new frontiers in cancer biology. J Biomech 47:1969–1978. 24300038 10.1016/j.jbiomech.2013.09.029

[cm21336-bib-0013] Hussain RH , Zawawi M , Bayfield MA. 2013 Conservation of RNA chaperone activity of the human La‐related proteins 4, 6 and 7. Nucleic Acids Res 41:8715–8725. 23887937 10.1093/nar/gkt649 PMC3794603

[cm21336-bib-0014] Ji X , Lu H , Zhou Q , Luo K. 2014 LARP7 suppresses P‐TEFb activity to inhibit breast cancer progression and metastasis. Elife 3:e02907. 25053741 10.7554/eLife.02907 PMC4126343

[cm21336-bib-0015] Kotik‐Kogan O , Valentine ER , Sanfelice D , Conte MR , Curry S. 2008 Structural analysis reveals conformational plasticity in the recognition of RNA 3′ ends by the human La protein. Structure 16:852–862. 18547518 10.1016/j.str.2008.02.021 PMC2430598

[cm21336-bib-0016] Kuspert M , Murakawa Y , Schaffler K , Vanselow JT , Wolf E , Juranek S , Schlosser A , Landthaler M , Fischer U. 2015 LARP4B is an AU‐rich sequence associated factor that promotes mRNA accumulation and translation. RNA 21:1294–1305. 26001795 10.1261/rna.051441.115 PMC4478348

[cm21336-bib-0017] Merret R , Martino L , Bousquet‐Antonelli C , Fneich S , Descombin J , Billey E , Conte MR , Deragon JM. 2013 The association of a La module with the PABP‐interacting motif PAM2 is a recurrent evolutionary process that led to the neofunctionalization of La‐related proteins. RNA 19:36–50. 23148093 10.1261/rna.035469.112 PMC3527725

[cm21336-bib-0018] Mura M , Hopkins TG , Michael T , Abd‐Latip N , Weir J , Aboagye E , Mauri F , Jameson C , Sturge J , Gabra H , et al. 2015 LARP1 post‐transcriptionally regulates mTOR and contributes to cancer progression. Oncogene 34:5025–5036. 25531318 10.1038/onc.2014.428 PMC4430325

[cm21336-bib-0019] Neve RM , Chin K , Fridlyand J , Yeh J , Baehner FL , Fevr T , Clark L , Bayani N , Coppe JP , Tong F , et al. 2006 A collection of breast cancer cell lines for the study of functionally distinct cancer subtypes. Cancer Cell 10:515–527. 17157791 10.1016/j.ccr.2006.10.008 PMC2730521

[cm21336-bib-0020] Nussinov R , Tsai CJ. 2015 'Latent drivers' expand the cancer mutational landscape. Curr Opin Struct Biol 32:25–32. 25661093 10.1016/j.sbi.2015.01.004

[cm21336-bib-0021] Pon JR , Marra MA. 2015 Driver and passenger mutations in cancer. Annu Rev Pathol 10:25–50. 25340638 10.1146/annurev-pathol-012414-040312

[cm21336-bib-0022] Ridley AJ. 2015 Rho GTPase signalling in cell migration. Curr Opin Cell Biol 36:103–112. 26363959 10.1016/j.ceb.2015.08.005 PMC4728192

[cm21336-bib-0023] Rohn JL , Sims D , Liu T , Fedorova M , Schock F , Dopie J , Vartiainen MK , Kiger AA , Perrimon N , Baum B. 2011 Comparative RNAi screening identifies a conserved core metazoan actinome by phenotype. J Cell Biol 194:789–805. 21893601 10.1083/jcb.201103168 PMC3171124

[cm21336-bib-0024] Sahai E. 2005 Mechanisms of cancer cell invasion. Curr Opin Genet Dev 15:87–96. 15661538 10.1016/j.gde.2004.12.002

[cm21336-bib-0025] Schaffler K , Schulz K , Hirmer A , Wiesner J , Grimm M , Sickmann A , Fischer U. 2010 A stimulatory role for the La‐related protein 4B in translation. RNA 16:1488–1499. 20573744 10.1261/rna.2146910 PMC2905749

[cm21336-bib-0026] Sommer G , Dittmann J , Kuehnert J , Reumann K , Schwartz PE , Will H , Coulter BL , Smith MT , Heise T. 2011a The RNA‐binding protein La contributes to cell proliferation and CCND1 expression. Oncogene 30:434–444. 20856207 10.1038/onc.2010.425

[cm21336-bib-0027] Sommer G , Rossa C , Chi AC , Neville BW , Heise T. 2011b Implication of RNA‐binding protein La in proliferation, migration and invasion of lymph node‐metastasized hypopharyngeal SCC cells. PLoS One 6:e25402. 22016766 10.1371/journal.pone.0025402 PMC3189910

[cm21336-bib-0028] Stavraka C , Blagden S. 2015 The La‐related proteins, a family with connections to cancer. Biomolecules 5:2701–2722. 26501340 10.3390/biom5042701 PMC4693254

[cm21336-bib-0029] Trotta R , Vignudelli T , Candini O , Intine RV , Pecorari L , Guerzoni C , Santilli G , Byrom MW , Goldoni S , Ford LP , et al. 2003 BCR/ABL activates mdm2 mRNA translation via the La antigen. Cancer Cell 3:145–160. 12620409 10.1016/s1535-6108(03)00020-5

[cm21336-bib-0030] Valderrama F , Thevapala S , Ridley AJ. 2012 Radixin regulates cell migration and cell‐cell adhesion through Rac1. J Cell Sci 125:3310–3319. 22467863 10.1242/jcs.094383

[cm21336-bib-0031] Vega FM , Fruhwirth G , Ng T , Ridley AJ. 2011 RhoA and RhoC have distinct roles in migration and invasion by acting through different targets. J Cell Biol 193:655–665. 21576392 10.1083/jcb.201011038 PMC3166870

[cm21336-bib-0032] Wurth L , Gebauer F. 2015 RNA‐binding proteins, multifaceted translational regulators in cancer. Biochim Biophys Acta 1849:881–886. 25316157 10.1016/j.bbagrm.2014.10.001

[cm21336-bib-0033] Yang R , Gaidamakov SA , Xie J , Lee J , Martino L , Kozlov G , Crawford AK , Russo AN , Conte MR , Gehring K , et al. 2011 La‐related protein 4 binds poly(A), interacts with the poly(A)‐binding protein MLLE domain via a variant PAM2w motif, and can promote mRNA stability. Mol Cell Biol 31:542–556. 21098120 10.1128/MCB.01162-10 PMC3028612

