# Supplementary material for: The RNA‐binding protein LARP4 regulates cancer cell migration and invasion
Source: Cytoskeleton (Hoboken). 2016 Sep 26;73(11):680–90. doi: 10.1002/cm.21336 (PMC5111583; doi:10.1002/cm.21336)
Supplement: Supplementary file 1 — Supporting Figures [file CM-73-680-s001.pdf]

Figure S1

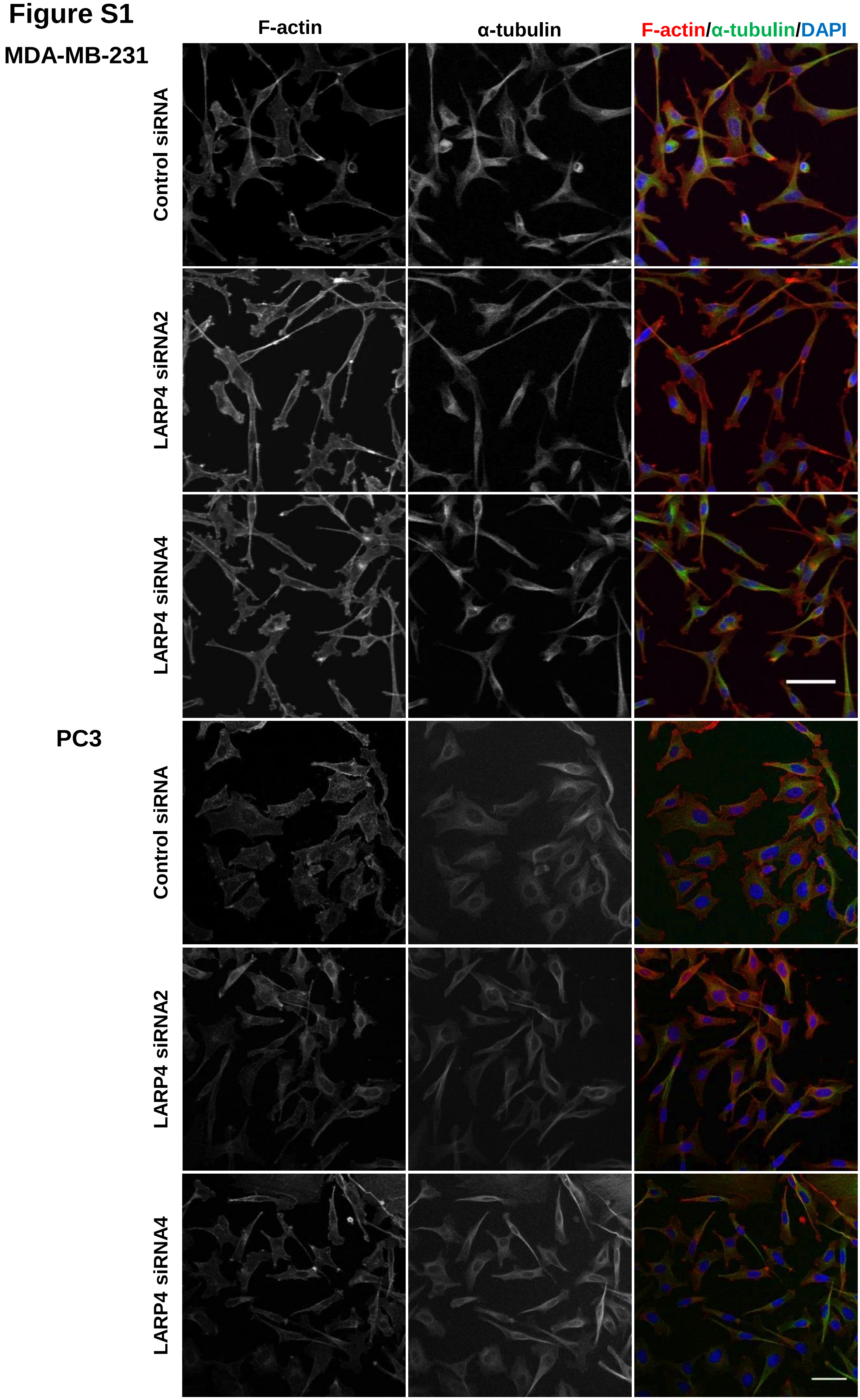

MDA-MB-231
PC3
F-actin
α-tubulin
F-actin/α-tubulin/DAPI
Control siRNA
LARP4 siRNA2
LARP4 siRNA4
Control siRNA
LARP4 siRNA2
LARP4 siRNA4

Figure S2

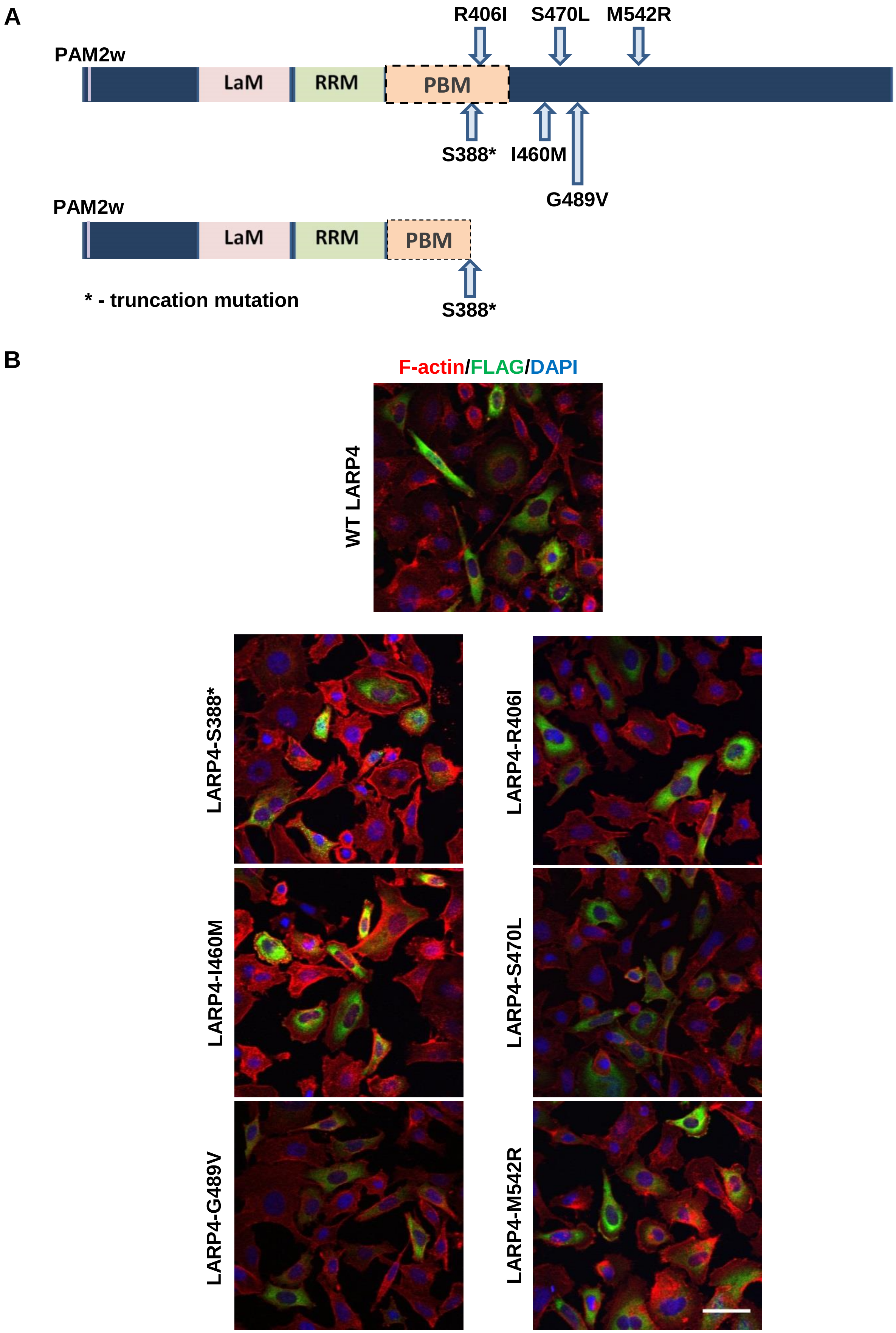
A
R406I
S470L
M542R
PAM2w
LaM
RRM
PBM
S388*
I460M
G489V
PAM2w
LaM
RRM
PBM
S388*
* - truncation mutation
B
F-actin/FLAG/DAPI
WT LARP4
LARP4-S388*
LARP4-R406I
LARP4-I460M
LARP4-S470L
LARP4-G489V
LARP4-M542R
