# Supplementary material for: The RNA‐binding protein LARP4 regulates cancer cell migration and invasion
Source: Cytoskeleton (Hoboken). 2016 Sep 26;73(11):680–90. doi: 10.1002/cm.21336 (PMC5111583; doi:10.1002/cm.21336)
Supplement: Supplementary file 2 — Supporting Legends [file CM-73-680-s002.docx]

**Supplementary material legends**

**Figure S1. LARP4 depletion alters cell shape**

MDA-MB-231 and PC3 cells were transfected with control siRNA or siRNAs targeting LARP4. Cells were fixed on coverslips and stained for F-actin (red), α-tubulin (green) and DNA (DAPI; blue). Scale bar: 50 µm. (A) Representative confocal images of LARP4-depleted MDA-MB-231 cells from one of the three independent experiments. (B) Representative confocal images of LARP4-depleted PC3 cells from one of the three independent experiments; images of α-tubulin are maximum intensity projections of 26 to 28 z-stacks.

**Figure S2. LARP4 mutations do not affect LARP4 localization**

(A) Schematic representation of the domain organisation of LARP4, showing the mutations, investigated in this study. R406I, I460M, S470L, G489V and M542R are point mutations; S388* encodes a stop codon instead of S388 and is therefore a truncation mutant of LARP4. PAM2w, PABP interaction motif 2 (variant with tryptophan, W); LaM, La motif; RRM, RNA recognition motif; PBM, putative PABP-binding motif. (B) PC3 cells were transfected with control (pEGFP-C1), wild-type LARP4 (WT) or LARP4 mutants. Cells were fixed on coverslips and stained for F-actin (red), FLAG epitope (green) and DNA (DAPI; blue). Representative images are from one of three independent experiments. Scale bar: 50 µm.

**Movie S1. Migration of LARP4-depleted MDA-MB-231 cells**

MDA-MB-231 cells were transfected with control siRNA or siRNAs targeting LARP4 and plated on collagen-coated wells, 48 hours after siRNA transfection. Images were acquired at 1 frame/10 min for 16 h. Movies were accelerated to 9.6 frames/s. Scale bar: 50 µm. (A) Cells transfected with control siRNA. (B) Cells transfected with siLARP4-2. (C) Cells transfected with siLARP4-4.

**Movie S2. Migration of LARP4-depleted PC3 cells**

PC3 cells were transfected with control siRNA or siRNAs targeting LARP4 and plated on Matrigel-coated wells, 24 h after siRNA transfection. After 31 h, images were acquired at 1 frame/10 min for 16 h. Movies were accelerated to 9.6 frames/s. Scale bar: 50 µm. (A) Cells transfected with control siRNA. (B) Cells transfected with siLARP4-2. (C) Cells transfected with siLARP4-4.
